# Preparation and *In Vivo* Evaluation of Indomethacin Loaded True Nanoemulsions

**DOI:** 10.3797/scipharm.0911-04

**Published:** 2009-11-23

**Authors:** Faiyaz Shakeel, Wafa Ramadan, Huda M. Gargum, Rajinder Singh

**Affiliations:** 1 Department of Pharmaceutics, Faculty of Pharmacy, Al-Arab Medical University, Benghazi-5341, Libya; 2 Department of Pharmacology, Faculty of Medicine, Al-Arab Medical University, Benghazi-5341, Libya; 3 Department of Pharmacognosy and Phytochemistry, Faculty of Pharmacy, Al-Arab Medical University, Benghazi-5341, Libya

**Keywords:** Anti-inflammatory effects, Skin irritation, Indomethacin, True nanoemulsions, Histopathology

## Abstract

Indomethacin, a potent nonsteroidal anti-inflammatory drug, has been used in the treatment of various kinds of pains, inflammation and arthritis. However, oral administration of indomethacin produces serious gastrointestinal adverse effects. Therefore the aim of the present investigation was to evaluate the anti-inflammatory effects, skin irritation, activation energy and histopathology of indomethacin from transdermally applied true nanoemulsion. The anti-inflammatory effects of true nanoemulsions were compared with marketed Indobene^®^ gel on carrageenan-induced paw edema in rats. Skin irritation tests were performed on Wistar rats for 14 days. The % inhibition value after 12 h application was significant for optimized formulation F6 (83) as compared to marketed Indobene^®^ gel (P<0.01). Results of skin irritation test indicated that developed true nanoemulsion is safe for human use. The significant decrease in activation energy (1.396 kcal/mol) for indomethacin across rat skin indicated that the stratum corneum lipid bilayers were significantly disrupted (P<0.05). From these results it was concluded that the developed nanoemulsion have great potential for transdermal application of indomethacin.

## Introduction

Indomethacin, a potent nonsteroidal anti-inflammatory drug (NSAID) has been used in the treatment of various kinds of pains, inflammation and arthritis [1, 2]. However oral administration of indomethacin produces serious gastrointestinal (GI) adverse effects upon chronic administration [2]. Therefore an alternative route is required to eliminate these oral adverse effects. Transdermal route has been known to eliminate oral GI adverse effects and maintains the plasma drug level for longer period of time and suitable for long treatment of chronic diseases like arthritis [3–8]. Therefore the aim of the present study was to evaluate the anti-inflammatory effects, skin irritation and skin permeation mechanism of indomethacin from transdermally applied true nanoemulsion in order to eliminate its GI adverse effects.

True nanoemulsions and oil-in-water (o/w) nanoemulsions are same systems and they are thermodynamically stable transparent isotropic dispersions of oil and water stabilized by an interfacial film of surfactant molecules having the droplet size less than 100 nm [8–10]. These systems are different from lipid nanoemulsions in terms of thermodynamic stability [10]. True nanoemulsions or nanoemulsions have been known to increase skin permeation, therapeutic efficacy and bioavailability of many drugs [4–15]. True nanoemulsions have been proved good vehicle for enhancement of anti-inflammatory effects (therapeutic efficacy) of some anti-inflammatory drugs [4, 7, 11]. Skin permeation mechanism of some anti-inflammatory drugs like celecoxib and aceclofenac using different techniques like fourier transform infra-red (FTIR), differential scanning calorimetry (DSC), activation energy determination and histopathological examination using true nanoemulsion has also been reported [7, 9]. Development, characterization and ex vivo skin permeation studies of indomethacin loaded true nanoemulsions have already been discussed in our previously published article [10]. Anti-inflammatory effects, skin irritation and skin permeation mechanism of indomethacin using true nanoemulsion have not been discussed in our previous article [10]. Moreover this in vivo evaluation (anti-inflammatory effects, skin irritation and skin permeation mechanism) has not been reported in the literature. Therefore in this article we evaluate the anti-inflammatory effects, skin irritation and skin permeation mechanism of indomethacin from transdermally applied nanoemulsion and compare its therapeutic effects with marketed Indobene^®^ gel. Nanoemulsions were prepared using nonirritant, pharmaceutically acceptable ingredients without using additional chemical enhancers.

## Results and discussion

**Fig. 1.**
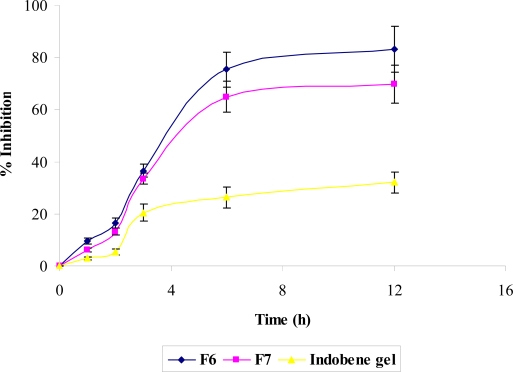
Anti-inflammatory effects of true nanoemulsion (F6), true nanoemulsion (F7) and marketed Indobene^®^ gel in Wistar male rats

True nanoemulsions of indomethacin were successfully prepared and characterized in terms of droplet size, viscosity, refractive index and *in vitro* skin permeation profile [10]. Based on the best skin permeation profile, lowest droplet size (25.53 & 34.64 nm for F6 and F7 respectively) and lowest viscosity (14.32 and 21.53 cp for F6 & F7 respectively), formulations F6 and F7 were selected for *in vivo* studies [10]. Anti-inflammatory effects of indomethacin in optimized formulations (F6 and F7) were evaluated to prove their therapeutic efficacy. Anti-inflammatory effects of developed nanoemulsions were also compared with marketed Indobene^®^ gel. The results of these studies are given in Table 1 and Figure 1. The % inhibition value after 12 h application was found to be highest for F6 (83) as compared to marketed Indobene^®^ gel (32.1 %). This difference was extremely significant at 1 % level of significance (P<0.01) (Figure 1). The % inhibition value was intermediate for formulation F7 (69.7). Initially (after 1, 2 and 3 h) % inhibition value was very low for both nanoemulsions. After 6 and 12 h there was significant enhancement in this value which indicated the sustained type of anti-inflammatory effects in formulations F6 and F7 (P<0.05). The anti-inflammatory effects of marketed Indobene^®^ gel were very poor after 1, 2, 3, 6 and 12 h application which indicated that marketed gel can be used only for topical or local drug delivery. On the other hand results of nanoemulsion formulations indicated that it can be used for local as well as for transdermal drug (systemic) delivery system (Figure 1 and Table 1). An increase in systemic anti-inflammatory effects of indomethacin leads to complete inhibition of the inflammation process [11]. Local gradient coupled with the increase in the amount of indomethacin that is absorbed over the period of 12 h caused almost complete inhibition of inflammation by nanoemulsion formulation.

The enhanced anti-inflammatory effects of true nanoemulsions could be due to the enhanced permeation of indomethacin through the skin [10].

Although all the materials used for preparation of nanoemulsion were falled under generally regarded as safe (GRAS) category. Concentration of all materials is very critical issue for these formulations. Large amount of surfactants is usually irritant to the skin. Therefore skin irritation tests were performed to confirm concentration of materials used for nanoemulsion preparation is safe. Moreover concentrations that we used in our formulations is beyond the limit that given in USFDA. Therefore these studies were necessary to confirm safety of developed formulation. Therefore skin irritation test was performed to confirm the safety of optimized true nanoemulsions of indomethacin. Van-Abbe et al. (1975) mentioned that a value between 0 and 9 indicates that the applied formulation is generally not irritant to human skin [16].

The mean values of skin irritation score for formulation F6 and F7 were found to be 1.66 ± 1.03 and 2.50 ± 0.54 respectively. From these results of 14 days test it was concluded that formulations F6 and F7 were safe to be used as transdermal drug delivery system (Table 2).

**Fig. 2.**
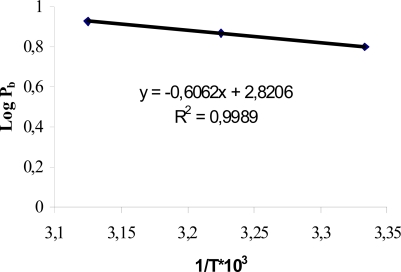
Arrhenius plot of true nanoemulsion F6 for determination of activation energy through rat skin

The value of activation energy (E_a_) could modify due to changes in skin composition and hence change the physicochemical properties of drug [7–9]. Nanoemulsions because of their action on stratum corneum (SC) lipids can change the value of E_a_. The Arrhenius plot between logarithms of permeability coefficient (log P_b_) and reciprocal of absolute temperature (1/T) was found linear in the temperature range of 27–47°C as shown in Figure 2, indicating no significant structural or phase transition changes within the skin membrane. The E_a_ value for permeation of indomethacin across rat skin was determined from the slope of Arrhenius plot (Figure 2). The E_a_ of indomethacin from true nanoemulsion F6 was found to be 1.396 kcal/mol. The activation energy for ion transport has been reported as 10.7 and 4.1 kcal/mol across phosphatidylcholine bilayer and human epidermis respectively [17, 18]. The significant decrease in E_a_ value for indomethacin permeation across rat skin indicated that the SC lipid bilayers were significantly disrupted (P<0.05).

In our study, E_a_ of indomethacin from formulation F6 was 1.396 kcal/mol. Therefore it was interpreted that nanoemulsions create pathways in the lipid bilayers of SC resulting in enhanced transdermal permeation of indomethacin [19].

**Fig. 3.**
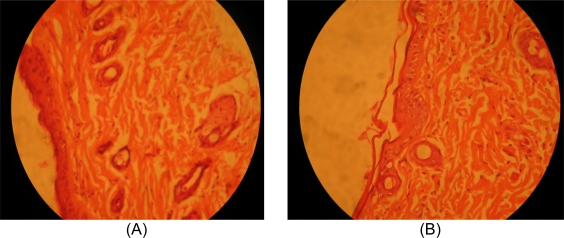
Photomicrograph of skin sample from (A) control group animal and (B) true nanoemulsion (F6) treated animal at low power (40 X)

Histopathological examination of nanoemulsion treated and control rat skin was performed using Carl Zeiss light microscope. The photomicrographs of control rat skin showed normal skin layers as shown in Figure 3A. When the skin was treated with nanoemulsion (F6) for 24 h, definite changes were observed in the skin morphology as shown in Figure 3B that could be due to the action of true nanoemulsion on stratum corneum. Dermis does not show any inflammatory cell infiltration (Figure 3B). There were no apparent signs of skin irritation (erythma and edema) observed on skin specimens treated with nanoemulsion F6 indicating the absence of any skin irritation as a consequence of true nanoemulsion treatment. These results indicated that developed nanoemulsion is safe for transdermal delivery of indomethacin.

## Conclusion

The *in vivo* anti-inflammatory studies revealed significant increase in % inhibition value of F6 as compared to marketed Indobene^®^ gel (P<0.05). This indicates that developed nanoemulsion formulation is efficacious. Results of skin irritation test indicated that formulation F6 is safe for human use. The significant decrease in activation energy for indomethacin indicates that the SC lipid bilayers was significantly disrupted (P<0.05). From these results it can be concluded that the developed nanoemulsion have great potential for transdermal application of indomethacin.

## Experimental

### Materials

Indomethacin was purchased from SAMA Impex (Berlin, Germany). Oleoyl macroglyceride (labrafil M1944CS) and diglycol monoethyl ether (transcutol-HP) were kind gift samples from Gattefossé (Cedex, France). Tween-80 was purchased from Sigma Aldrich (St. Louis, MO, USA). Marketed indomethacin gel (Indobene^®^) was purchased from local market (Benghazi, Libya). All other chemicals used were of analytical reagent (AR) grade.

### Preparation of true nanoemulsions

Various true nanoemulsions of indomethacin were prepared by spontaneous emulsification method (aqueous phase titration method). Two optimized nanoemulsion (F6 and F7) were selected for *in vivo* studies [10]. Selection of these optimized formulations was based on the best *in vitro* skin permeation profile, lowest viscosity, least polydispersity index, lowest droplet size and optimum surfactant concentration [10]. These true nanoemulsions were prepared by dissolving 0.5 % w/w of indomethacin in specified quantity of cosurfactant (transcutol-HP). Then surfactant (Tween-80) and oil (labrafil M1944CS) were added slowly in the oil phase. Then sufficient quantity of distilled water was added to get the final preparation 100 % w/w. The composition of these true nanoemulsions is given in Table 3.

### In vivo anti-inflammatory effects

Approval to carry out *in vivo* anti-inflammatory studies was obtained from the Local Animal Ethics Committee, Al-Arab Medical University, Benghazi, Libya and their guidelines were followed for the studies. The sustained anti-inflammatory effects of the optimized formulations (F6 and F7) were evaluated by carrageenan-induced hind paw edema method developed in Wistar rats by Winter et al. 1965 [20]. Young male Wistar rats weighing 200–250 g were randomly divided into 4 groups: control, true nanoemulsion (F6), true nanoemulsion (F7) and marketed indometacin (Indobene^®^) gel each containing 6 rats. The animals were kept under standard laboratory conditions, at a temperature of 25 ± 1°C and relative humidity of 55 ± 5%. The animals were housed in polypropylene cages, with free access to standard laboratory diet (Lipton feed, Tripoli, Libya) and water *ad libitum*. Dose for the rats was calculated based on the weight of the rats according to the surface area ratio [7, 8]. The dorsal side of the rats was shaved 12 h before starting the experiments except in control group. Formulations F6, F7 and Indobene^®^ gel were applied on the shaved dorsal region of all animals (except in control group) half an hour before subplanter injection of carrageenan in right paw. Paw edema was induced by injecting 0.1 ml of 1 % w/v dispersion of carrageenan in distilled water. The volume of paw was measured at 1, 2, 3, 6, and 12 h after injection using digital plethysmometer (Ugo Basile, Italy).

The amount of paw swelling was determined time to time and expressed as percent edema. Percent inhibition of edema produced by each formulation-treated group was calculated against the respective control group.

Results of these studies were compared using Dunnett test of one-way analysis of variance (ANOVA).

### Skin irritation test

Skin irritation test was performed on developed formulations F6 and F7 on male Wistar rats weighing 200–250 g. Wistar rats were divided into 2 groups: true nanoemulsion (F6) and true nanoemulsion (F7) each contain 6 rats. The animals were kept under standard laboratory conditions, temperature at 25 ± 1°C and relative humidity (55 ± 5%). The animals were housed in polypropylene cages, six per cage, with free access to standard laboratory diet (Lipton feed, Tripoli, Libya) and water *ad libitum*. A single dose of 10 μl of each optimized nanoemulsion formulation (F6 and F7) was applied to the left ear of the rat with the right ear as a control. The development of erythema was monitored for 14 days using the reported method of Van-Abbe et al. [16].

### Determination of activation energy

For determination of activation energy of indomethacin, *in vitro* skin permeation studies across rat skin were carried out at 27, 37 and 47°C in the vehicle methanolic phosphate buffer saline (PBS pH 7.4) (1:9) [7–9]. In the donor compartment, 1 ml of formulation was taken (containing 5 mg of indomethacin). Receiver compartment was composed of the vehicle methanolic PBS only. Permeability coefficients were determined at each temperature and activation energy of indomethacin was then calculated from Arrhenius equation [7–9].

$$\text{P}\;{=\;\text{P}}_{\text{o}}\;{\text{e}}^{-\text{Ea}/\text{RT}}$$

Where E_a_ is the activation energy, R is gas constant (1.987 kcal/mol), T is absolute temperature (K), P is the permeability coefficient and P_o_ is the Arrhenius factor.

### Histopathological examination of skin specimens

Dorsal skin of Wistar rat was treated with optimized true nanoemulsion (F6) in methanolic PBS pH 7.4. After 24 h, rat was sacrificed and the skin samples from treated and untreated (control) area were taken. Each skin sample was stored in 10 % formalin solution in methanolic PBS (pH 7.4). The skin samples were cut into vertically in different sections. Each section was dehydrated using ethanol, embedded in paraffin for fixing and stained with xylene. Skin samples were then observed under light microscope (Axioskop 40 FL, Carl Zeiss, Germany)) fitted with canon power shot G3 digital camera and compared with control sample. The light microscopy was performed at low magnification level (40X) [21, 22].

## Figures and Tables

**Tab. 1.** Anti-inflammatory effects of F6, F7 and Indobene^®^ gel in carrageenan-induced rat paw edema

**Group**	**Formulation**	**N**	**Mean Wt± SD (g)**	**Time (h)**	**Mean % Edema ± SD**	**% inhibition**
I	Control (carrageenan only)	6	210.0±22.5	1	27.2±3.1	
				2	40.0±4.1	
				3	74.5±5.7	
				6	57.0±4.2	
				12	38.3±3.3	
II	F6	6	220±25.5	1	24.6±2.4	9.5
				2	33.4±2.8	16.5
				3	47.3±4.1	36.5
				6	14.0±1.8	75.4
				12	6.5±1.2	83.0
III	F7	6	240.0±32.0	1	25.5±2.9	6.2
				2	34.7±3.2	13.2
				3	49.5±4.8	33.5
				6	20.0±2.9	64.9
				12	11.6±1.4	69.7
IV	Indobene^®^ gel	6	245.0±35.5	1	26.4±3.7	2.9
				2	37.8±4.9	5.5
				3	59.3±5.7	20.4
				6	42.0±4.5	26.3
				12	26.0±2.6	32.1

N = Number of rats in each group; SD = Standard deviation

**Tab. 2.** Skin irritation score of the true nanoemulsions F6 and F7

**S.No**	**Group**	**Score after (days)**						**Mean score± SD**
		**1**	**2**	**3**	**4**	**7**	**14**	

1	I (F6)	2	0	3	2	1	2	1.66± 1.03
2	II (F7)	3	2	2	3	3	2	2.50± 0.54

**Tab. 3.** Compositions of true nanoemulsion F6 and F7

**Ingredients (% w/w)**	**F6**	**F7**
Indomethacin	0.5	0.5
Labrafil M1944CS	5.0	10.0
Tween-80	33.75	45.0
Transcutol-HP	11.25	15.0
Distilled water (q.s.)	100.0	100.0

q.s. = Quantity sufficient to produce total formulation 100 % w/w
